# Diversity of culturable aerobic denitrifying bacteria in the sediment, water and biofilms in Liangshui River of Beijing, China

**DOI:** 10.1038/s41598-017-09556-9

**Published:** 2017-08-30

**Authors:** Pengyi Lv, Jinxue Luo, Xuliang Zhuang, Dongqing Zhang, Zhanbin Huang, Zhihui Bai

**Affiliations:** 1 School of Chemical and Environmental Engineering, China University of Mining and Technology (Beijing), Beijing, 100083 China; 2 Research Center for Eco-Environmental Sciences, Chinese Academy of Sciences, Beijing, 100085 China; 3 College of Resources and Environment, University of Chinese Academy of Sciences, Beijing, 100049 China; 4 Advanced Environmental Biotechnology Centre, Nanyang Environment and Water Research Institute, Nanyang Technological University, Singapore, 637141 Singapore

## Abstract

Aerobic denitrification is a process reducing the nitrate into gaseous nitrogen forms in the presence of oxygen gas, which makes the nitrification and denitrification performed simultaneously. However, little was known on the diversity of the culturable aerobic denitrifying bacteria in the surface water system. In this study, 116 strains of aerobic denitrifying bacteria were isolated from the sediment, water and biofilm samples in Liangshui River of Beijing. These bacteria were classified into 14 genera based on the 16 S rDNA, such as *Pseudomonas*, *Rheinheimera*, and *Gemmobacter*. The *Pseudomonas* sp., represented by the *Pseudomonas stutzeri*, *Pseudomonas mendocina* and *Pseudomonas putida*, composed the major culturable aerobic denitrifiers of the river, followed by *Ochrobactrum* sp. and *Rheinheimera* sp. The PCA plot showed the unclassified *Pseudomonas* sp. and *Rheinheimera pacifica* preferred to inhabit in biofilm phase while one unclassified *Ochrobactrum* sp. and *Pseudomonas resinovorans* had higher abundance in the sediment. In the overlying water, the *Pseudomonas stutzeri* and *Ochrobactrum rhizosphaerae* were found to have higher abundance, indicating these aerobic denitrifiers had different habitat-preferable characteristics among the 3 phases of river system. The findings may help select the niche to isolate the aerobic denitrifiers and facilitate the bioaugmentation-based purification of the nitrate polluted surface water.

## Introduction

The pollution of nitrate in surface water has increasingly become a serious environmental problem in modern world due to the increasing anthropogenic activities, such as discharge of industrial and domestic sewage, leaching of animal manure from the farming industry^1^ and excessive utilization of fertilizers in agriculture^2^. One significant concern of the nitrate pollution is eutrophication^3, 4^, which is represented by the thriving of aquatic plants and algae in water, resulting in the oxygen depletion and death of aquatic animals^5^. Besides, the accumulation of nitrate in water also increases the health risk to human. It was reported that high concentrations of nitrate (e.g. 10 mg/L) in the drinking water may increase the risk of the methaemoglobinaemia to human, a disease also named as Blue Baby Syndrome^6^. Furthermore, high levels of nitrate in drinking water was also showed positive associations to the occurrence of cancer, e.g. stomach cancer^7^ and gastric cancer^8^. Though much effort has been done to purify the polluted water, the level of nitrate in the surface water was still high according to the recent studies. In an investigation in Osona region of northeast Spain, approximately 70% of wells tested contained at least 50 mg/L of nitrate^9^. In North China Plain, the level of nitrate pollution ranges from 0.9 mg/L to 131 mg/L in the surface water^10^. Therefore, efficient methods are needed urgently to purify the nitrate contaminated surface water.

Traditionally, the nitrate removal is performed through a heterotrophically anaerobic denitrification (ANDN) process, where the nitrate is reduced to gaseous nitrogen forms in the absence of oxygen gas, such as the nitrous oxide (N_2_O), nitric oxide (NO) and dinitrogen gas (N_2_)^11^. However, there is a problem in the ANDN process, which is the exclusion of oxygen. Oxygen is a crucial factor affecting the transformation of nitrogen in water, e.g. the oxidation of ammonia and reduction of nitrate. Ammonia is another toxic nitrogen contaminant in surface water, which can be removed through being oxidized to nitrate (nitrification) in the presence of oxygen gas^12^. So the different requirement of oxygen determines the aerobic nitrification and anaerobic denitrification processes cannot be operated under the same condition. It is known that the surface water in nature, e.g. flowing river, is often an aerobic environment^13^. The oxygen scarcity often occurs when the eutrophication happens^3^, which is a situation needed to be avoided. Thus, the ANDN process is not suitable for the water purification *in situ* before the concentration of the contaminated nitrate exceeds the threshold and leads to the occurrence of eutrophication. Furthermore, many evidences indicated that the denitrification also occurred under the aerobic conditions^14^. Just as its name implies, the aerobic denitrification is a process which can simultaneously use the both oxygen and nitrate as the electron acceptors and therefore is capable of reducing the nitrate in the presence of oxygen gas^15^. Therefore, some researchers tried to isolate and apply the aerobic denitrifying bacteria in the removal of nitrate through the aerobic denitrification process^16^.

Some aerobic denitrifying bacteria have been isolated from different niches, such as the *Bacillus methylotrophicus* L7 isolated from wastewater^17^, *Pseudomonas putida* AD-21 isolated from soil^18^, *Pseudomonas stutzeri* TR2 isolated from domestic wastewater^19^ and *Zoogloea* sp. N299 isolated from the drinking water reservoir^20^. However, little is known on the community diversity of the culturable aerobic denitrifying bacteria in the surface water system. Therefore, in this study, the aerobic denitrifying bacterial strains were isolated from 3 types of niches in the Liangshui River, including the overlying water where the bacteria live in planktonic mode, biofilm phase where the microorganisms live in a sessile mode and sediment where high concentrations of nutrients, e.g. nitrogen and phosphorus, were usually deposited and absorbed from the overlying water phase^21, 22^. The classification and compositions of the aerobic denitrifying consortiums were also studied.

## Results

### Screening of the aerobic denitrifying bacteria from the river

Three types of the samples, including the overlying water, sediment and biofilms on supporting materials, were collected from the Liangshui River which is the receiving water of the effluents of Xiaohongmen wastewater treatment plant. The sampling points were locating at 500 m downstream of the outfall point of the wastewater treatment plant (Fig. S1). Samples of Liangshui River were collected in March 2015 at three niches: overlying water (at 50 cm depth), sediment (at 100 cm depth) and biofilm (at 50 cm depth). The average nitrogen concentrations in forms of ammonia and nitrate in sampling sites were tested to be 24.5 mg/L and 18.6 mg/L respectively, and the range of dissolved oxygen level was 1–4 mg/L. After the screening, a total of 116 strains of bacteria were tested to have the denitrifying ability under the aerobic conditions, all of which showed the positive reaction with the BTB (Fig. S2). An OTU based analysis of the 16S rDNA was conducted. At cutoff of 0.03, which is generally considered as the label for Species^23^, a total of 24 OTUs were clustered (Supplementary Table S1). The classification of the OTUs against to the RDP and NCBI 16S ribosome RNA reference databases was shown in Table 1. The rarefaction curve and OTU coverage analysis indicated that the isolated bacterial strains covered approximately 80–90% of the culturable bacterial community in each of the three niches, implying the data here were representative.

**Table 1:** The classification for the OTUs of culturable aerobic denitrifiers in surface water against to the NCBI 16S ribosome RNA reference database.

OTUs	Representative isolate	Taxonomic classification		
		Order	Genus	Species
OTU01	W19	Pseudomonadales	*Pseudomonas*	*mendocina*
OTU02	B11	Pseudomonadales	*Pseudomonas*	*stutzeri*
OTU03	B10	Pseudomonadales	*Pseudomonas*	*putida/monteilii*
OTU04	B12	Rhizobiales	*Ochrobactrum*	unclassified
OTU05	B33	Chromatiales	*Rheinheimera*	*pacifica*
OTU06	S11	Pseudomonadales	*Pseudomonas*	*resinovorans*
OTU07	W46	Rhizobiales	*Ochrobactrum*	*rhizosphaerae*
OTU08	W38	Rhodobacterales	*Pannonibacter*	*Phragmitetus*
OTU09	B23	Pseudomonadales	*Pseudomonas*	unclassified
OTU10	B38	Rhizobiales	*Rhizobium*	*pusense*
OTU11	W9	Actinobacteridae	*Gordonia*	*malaquae*
OTU12	S3	Actinobacteridae	*Gordonia*	*alkanivorans*
OTU13	W4	Xanthomonadales	*Stenotrophomonas*	*acidaminiphila*
OTU14	S20	Caulobacterales	*Brevundimonas*	*diminuta*
OTU15	W3	Actinobacteridae	*Gordonia*	*terrae*
OTU16	W5	Rhodobacterales	*Paracoccus*	*versutus*
OTU17	W2	Burkholderiales	*Comamonas*	*terrigena*
OTU18	S6	Actinobacteridae	*Rhodococcus*	*canchipurensis*
OTU19	W14	Rhizobiales	*Pseudochrobactrum*	*saccharolyticum*
OTU20	W8	Xanthomonadales	*Stenotrophomonas*	*terrae*
OTU21	W10	Actinobacteridae	*Arthrobacter*	*ureafaciens*
OTU22	B8	Rhodobacterales	*Gemmobacter*	*caeni*
OTU23	B7	Actinobacteridae	*Rhodococcus*	*Pyridinivorans*
OTU24	B5	Actinobacteridae	*Arthrobacter*	*soil*

**Figure 1:**
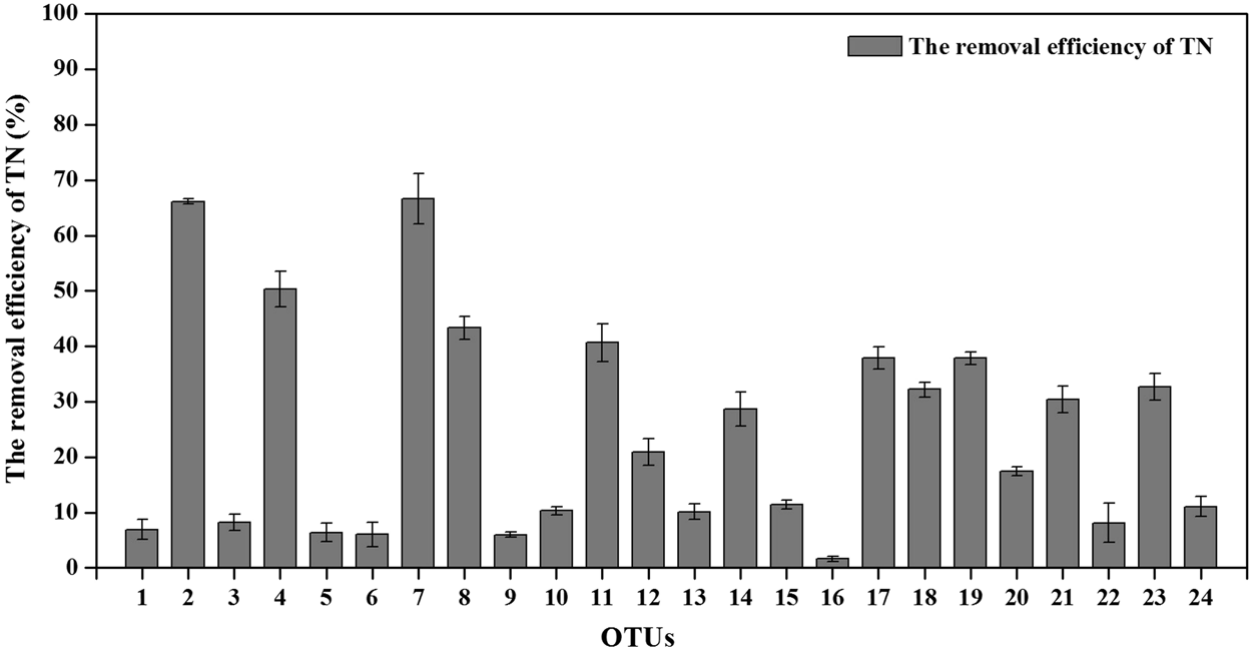
The removal efficiency of total nitrogen (TN) for the representative strains of the isolated 24 OTUs. The removal efficiency reflected the aerobic denitrification capacity.

The capability of total nitrogen removal was also tested through the incubation in the nitrate enriched medium (DM medium in this study) aerobically for these OTUs. The results showed that all the isolated OTUs had the aerobic denitrifying ability, where the removal efficiency of total nitrogen (TN) was in the range of 1.6–67% (Fig. 1). The top three aerobic denitrifying bacteria were OTU 7 (*O. rhizosphaerae*), OTU 2 (*Pm. stutzeri*) and OTU 4 (unclassified *Ochrobactrum* sp.), which reduced the TN by 66.63%, 66.17%, 50.30% respectively after the incubation for 96 hours. The TN removal efficiency of the rest OTUs were in the range of 6–50%, except for the OTU 16 (*Pa. versutus*) which had the TN reduction efficiency of 1.6%.

### Community of culturable aerobic denitrifiers in the overlying water

**Figure 2:**
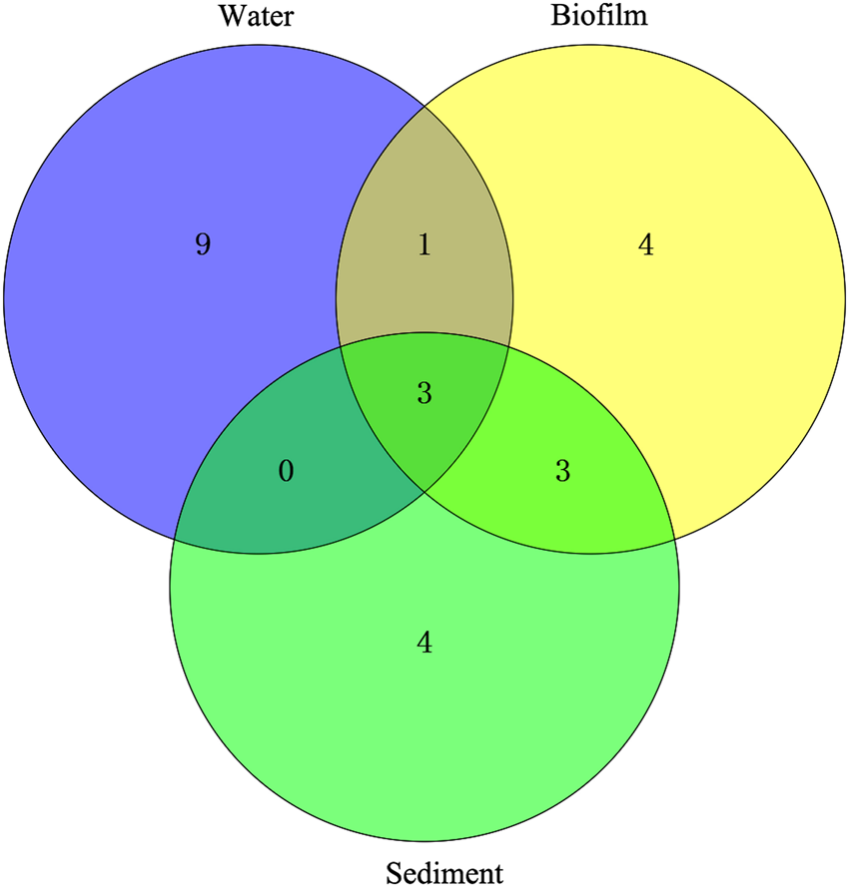
The Venn diagram of OTUs at cutoff of 0.03 for the culturable aerobic denitrifying bacteria in the phases of biofilm, water and sediment. The numbers in the overlapping parts represent the shared OTUs in different phases.

Among the 24 OTUs, 13 OTUs were isolated from the overlying water, 11 OTUs were isolated from the biofilms and 10 OTUs were isolated from the sediment phase samples (Fig. 2 and Supplementary Table S1). Therefore, the community structure was studied specifically in the three niches.

**Figure 3:**
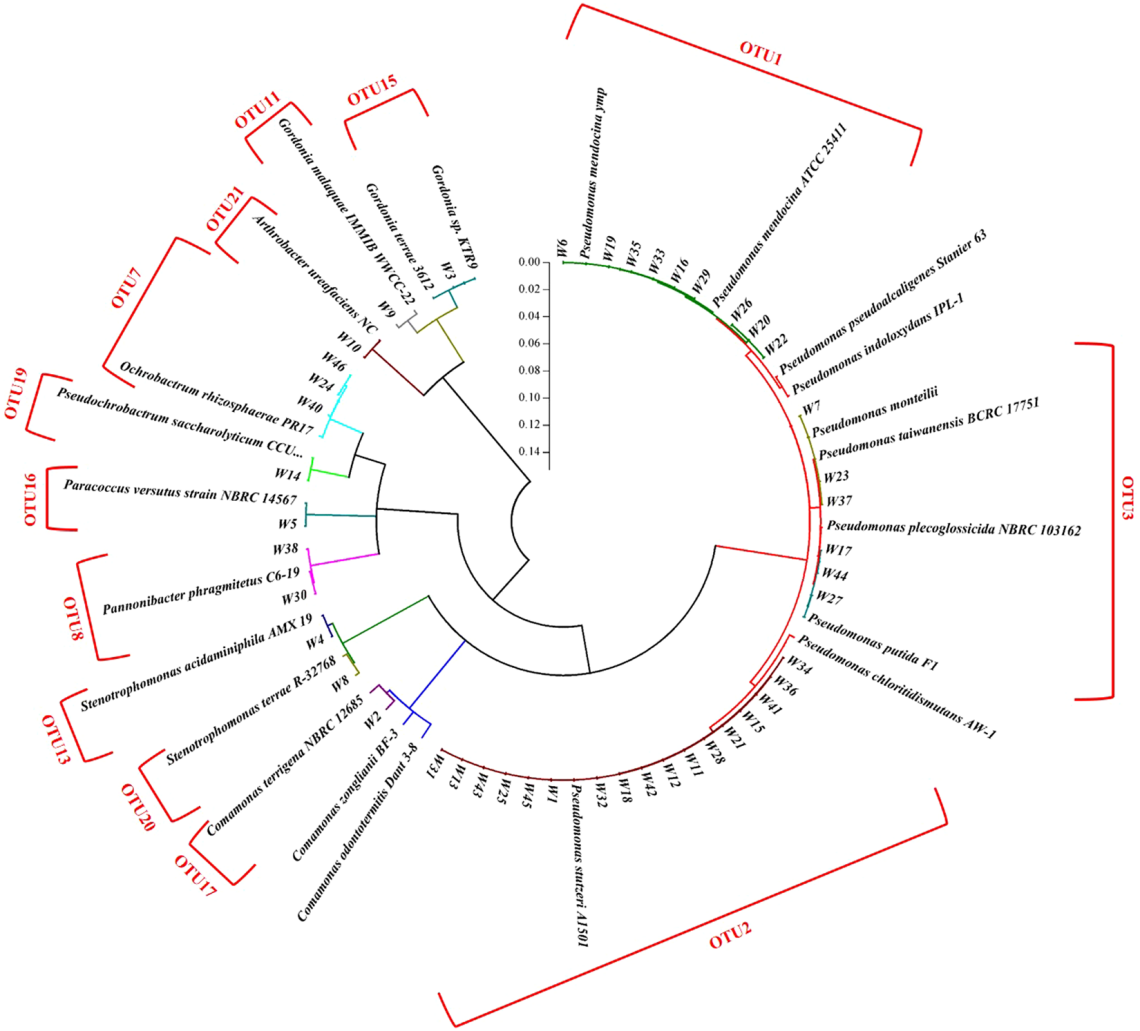
The phylogenetic tree (circular mode) for the culturable aerobic denitrifying isolates in the water phase of Liangshui River. The vertical scale bar in the middle represents the distance (dissimilarity) of the isolates and reference strains.

In the overlying water samples, all the 13 OTUs were composed of a group of 45 strains of bacteria (Fig. 3). After the sequence assignment against to the RDP and NCBI databases, they were clustered to 9 genera of bacteria, including *Pseudomonas* (*Pm*) sp., *Comamonas* sp., *Stenotrophomonas* sp., *Pannonibacter* (*Pn*) sp., *Paracoccus* sp., *Pseudocrobactrum* (*Pc*) sp., *Ochrobactrum* sp., *Arthrobacter* sp. and *Gordonia* sp. (Supplementary Fig. S3). In the genus of *Pseudomonas*, 17 strains of isolates, such as W1, W15, W36, W45, etc. were assigned to the *Pm. stutzeri*, 9 strains of isolates, including W6, W16, W35, etc. were classified to the *Pm. mendocina* (Fig. 3). In the genus of *Gordonia*, the strain W3 was classified to *G. terrae* while the W9 was assigned to *G. malaquae* (Fig. 3). In addition, in the overlying water, the culturable aerobic denitrifiers also included *Pn. phragmitetus* (W30 and W38), *Pc. saccharolyticum* (W14), *O. rhizosphaerae* (W24, W40 and W46), *A. ureafaciens* (W10), *S. terrae* (W8), *S. acidaminiphila* (W4), and unclassified *Comamonas* sp. (W2).

### Community of culturable aerobic denitrifiers in the biofilm

**Figure 4:**
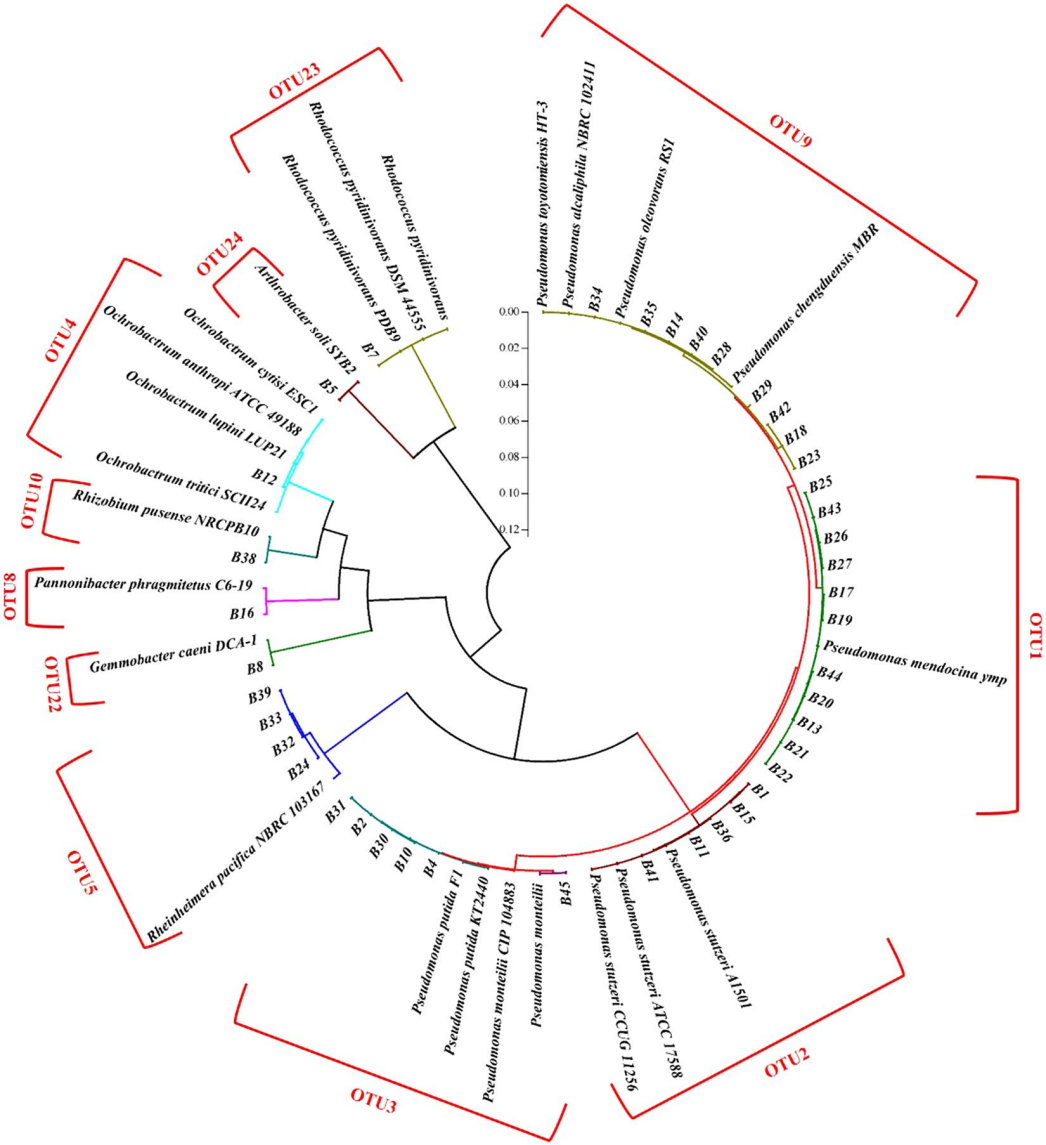
The phylogenetic tree (circular mode) for the culturable aerobic denitrifying isolates from the biofilm phase of Liangshui River. The vertical scale bar in the middle represents the distance (dissimilarity) of the isolates and reference strains.

In this study, 11 OTUs, composed of a group of 41 strains of bacteria, were isolated from the biofilm samples and screened to have the denitrifying ability under the aerobic condition (Fig. 4). Against to the RDP and NCBI databases, 8 genera of bacteria were assigned, including the *Pseudomonas* (*Pm*) sp., *Rheinheimera* (*Rh*) sp., *Gemmobacter* sp., *Pannonibacter* (*Pn*) sp., *Rhizobium* (*Rz*) sp., *Ochrobactrum* sp., *Arthrobacter* sp. and *Rhodococcus* (*Rc*) sp. (Supplementary Fig. S4). The *Pseudomonas* sp. were the most abundant culturable aerobic denitrifiers in the biofilm phase, which were composed of 11 strains of *Pm. Mendocina* (B17, B19, B44, etc.), 1 strain of *Pm. monteilii* (B45), 5 strains of *Pm. putida* (B4, B10, B30, etc.), 5 strains of *Pm. stutzeri* (B1, B15, B36, etc.) and 9 strains of unclassified *Pseudomonas* sp. (B34, B40, B42, etc.) that all had a very close relation with the *Pm. toyoyomensis* HT-3, *Pm. Oleovorans* RS1 and *Pm. chengduensis* MBR (Fig. 4). The *Rheinheimera* were the second abundant genus which all were hit to the *Rh. pacifica* (B24, B32, B33, B39). In addition, 4 species including *G. caeni* (B8), *Pn. phragmitetus* (B16), *Rz. pusense* (B38), *A. soil* (B5) and *Rc. Pyridinivorans* (B7) were also isolated. The strain B12 had the same similarity (99.85%) to each of the *O. anthropi* ATCC 49188, *O. lupine* LUP21 and *O. cytisi* ESC1, which was therefore cannot be identified at the Species level just based on the 16S rDNA.

### Community of culturable aerobic denitrifiers in the sediment

**Figure 5:**
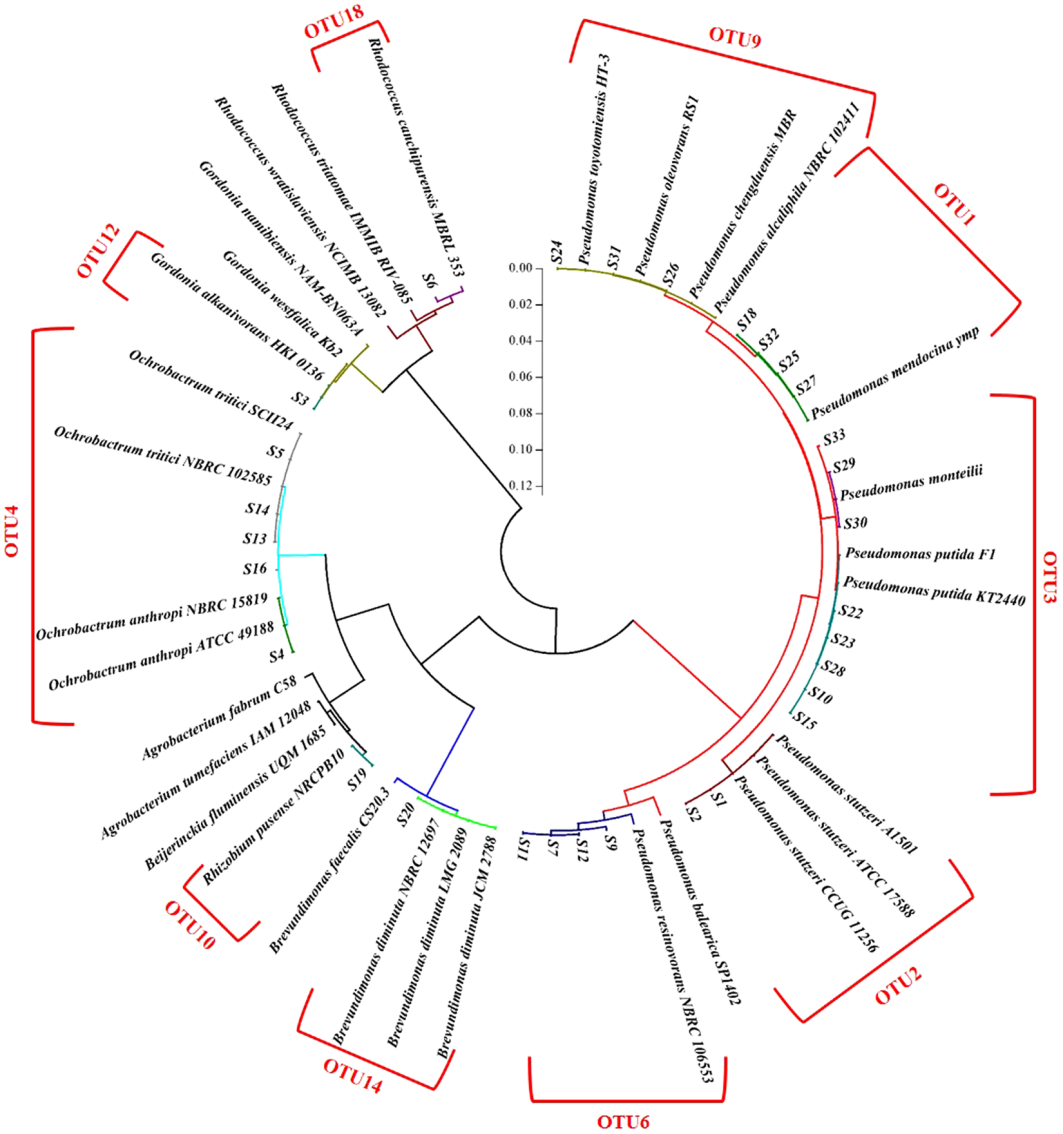
The phylogenetic tree (circular mode) for the culturable aerobic denitrifying isolates from the sediment phase of Liangshui River. The vertical scale bar in the middle represents the distance (dissimilarity) of the isolates and reference strains.

The sediment in the river is often composed of the soil, sands, deteriorated materials and the benthic microbial communities^24^. The nutrient levels are usually very high in the sediment, due to the deposition and absorption of the organics and inorganics from the overlying water phase^21, 22^. On the other hand, the sediment is also a reservoir which released the nutrient and microorganisms to the overlying water phase. Therefore, the aerobic denitrifying bacteria were also isolated from the sediment phase in Liangshui River. In total, 10 OTUs which were composed of 30 strains of aerobic denitrifying bacteria were acquired from the sediment. These OTUs can be grouped into 6 genera, including the *Pseudomonas* (*Pm*) sp. *Brevundimonas* sp., *Rhizobium* (*Rz*) sp., *Ochrobactrum* sp., *Gordonia* sp. and *Rhodococcus* (*Rc*) sp. (Fig. 5 and Supplementary Fig. S5). Similarly with the phases of water and biofilm, the Genus *Pseudomonas* dominated the whole culturable aerobic denitrifying community, which contained 5 strains of *Pm. putida* (S10, S15, S22, etc.), 4 strains of *Pm. mendocina* (S18, S25, S27 and S32), 4 strains of *Pm. resinovorans* (S7, S9, S11 and S12), 2 strains of *Pm. stutzeri* (S1 and S2), 3 strains of *Pm. monteilii* (S29, S30 and S33) and 3 strains of unclassified *Pseudomonas* sp. (S24, S26 and S31). The Genus *Ochrobactrum* were the second abundant culturable aerobic denitrifiers in sediment, which included 4 strains of *O. tritici* (S5, S13, S14 and S16) and 1 strain of *O. anthropi* (S4). In addition, some rare species were also isolated, including *B. diminuta* (S20), *Rz. pusense* (S19), *G. alkanivorans* (S3) and *Rc. canchipurensis* (S6).

### Distribution of aerobic denitrifiers in overlying water, biofilm and sediment

**Figure 6:**
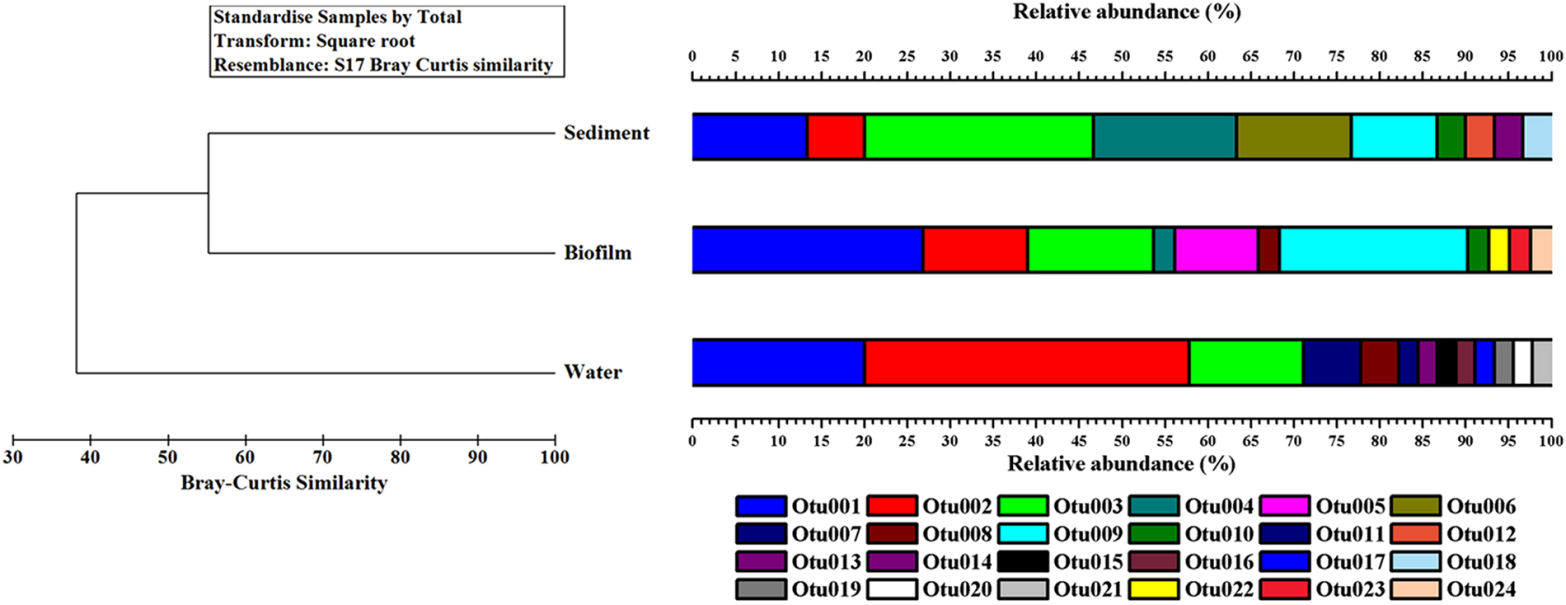
The OTU compositions and relationship of aerobic denitrifying bacteria in biofilm, water and sediment. The clustering tree on the left half was constructed based on the Bray-Curtis similarity of OTUs compositions which is on the right half of the figure, in which cluster 1 was the combination of biofilm and sediment and cluster 2 included cluster 1 and water.

**Figure 7:**
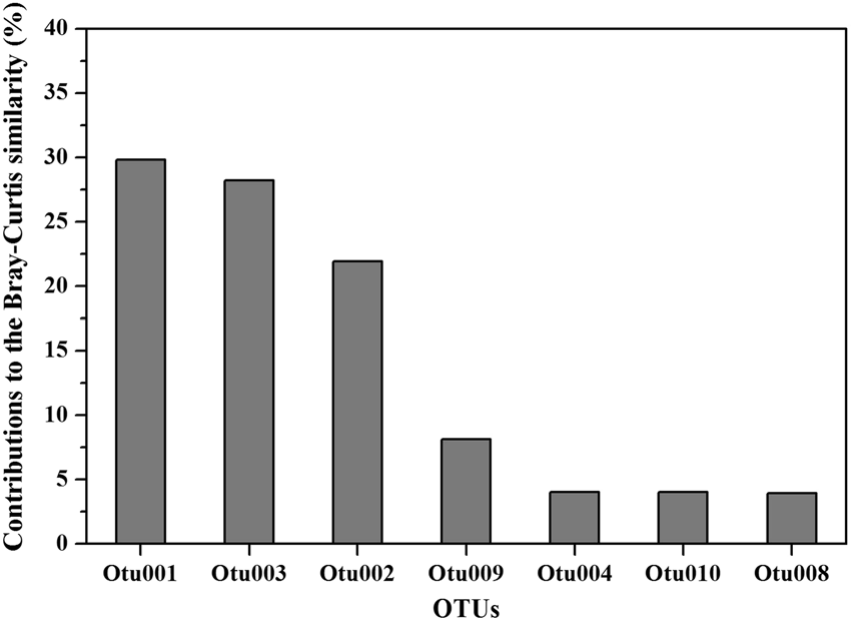
The contributions of different OTUs to the Bray-Curtis similarity of culturable aerobic denitrifiers in the phases of water, biofilm and sediment. The values of contributions were calculated in the PRIMER 6.0.

In order to compare the habitat-preference of the culturable aerobic denitrifiers, the beta-diversity of the communities was studied between the overlying water, biofilm and sediment. Among them, the biofilm and sediment samples shared the highest number of OTUs (6 OTUs), in comparison with the group of biofilm and water (4 OTUs) and group of sediment and water (3 OTUs) (Fig. 2). Only 3 OTUs were found in all the 3 phases of samples, which included OTU 1 (*Pm. mendocina*), OTU 2 (*Pm. stutzeri*) and OTU 3 (*Pm. putida*). Examined by the cluster analysis, the culturable aerobic denitrifying communities had only 38.1% of Bray-Curtis similarity in the phases of water, biofilm and sediment. Therein, the communities of biofilm and sediment had a closer relationship (55.2% in Bray-Curtis similarity) relative to the aerobic denitrifying bacteria in water (Fig. 6). Examined through the “SIMPER” analysis by the PRIMER 6.0, the *Pm. mendocina* contributed most to the Bray-Curtis similarity (29.8% in contribution), followed by the *Pm. putida* (28.2% in contribution) and *Pm. stutzeri* (21.9% in contribution), which together contributed 79.9% to the Bray-Curtis similarity (Fig. 7). This implied these three species may be the universal aerobic denitrifiers in the phases of overlying water, biofilm and sediment of the Liangshui River system.

**Figure 8:**
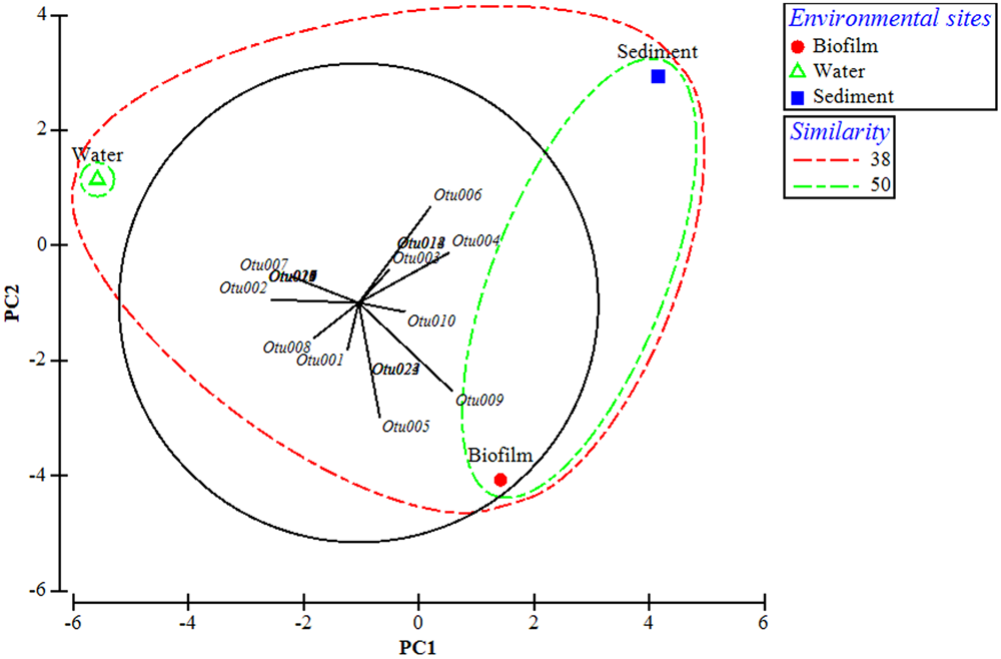
The PCA plot and dominant OTUs corresponding to the dissimilarity of aerobic denitrifying bacterial communities in the phases of biofilm, water and sediment. The red circle, blue square and green triangle represent the culturable communities in biofilm, sediment and water respectively. The black branches represent the dominant OTUs which contributed most to the dissimilarity among the communities in the 3 environmental sites. The blank circle indicating a maximal vector which contains all the base variables, and it is a default setting in PRIMER 6.0.

On the other hand, the culturable aerobic denitrifiers presented 61.9% of dissimilarity among the phases of overlying water, biofilm and sediment. The PCA plot indicated that the OTU 5 (*Rh. pacifica*) and OTU 9 (unclassified *Pseudomonas* sp.) preferred to inhabit in biofilms rather than the water and sediment (Fig. 8). For example, the OTU 9 (unclassified *Pseudomonas* sp.) accounted for 22.0% and 10% of the culturable aerobic denitrifiers in biofilms and sediment respectively, but no strains of OTU 9 were isolated from the phase of water (Fig. 6). Especially, the OTU 5 (*Rh. pacifica*) was only isolated from the biofilm phase (Fig. 6). In the sediment, OTU 4 (unclassified *Ochrobactrum* sp.) and OTU 6 (*Pm. resinovorans*) contributed most to the dissimilarity between the sediment phase and the other two phases (Fig. 8). In addition, OTU 12 (*G. alkanivorans*), OTU 14 (*B. diminuta*) and OTU 18 (*Rc. canchipurensis*) were only isolated from the sediment, which all had the relative abundance of 3.3% in the sediment community. Examined by the PCA plot, among the OTUs in water phase, the OTU 2 (*Pm. stutzeri*) and OTU 7 (*O. rhizosphaerae*) contributed most to the dissimilarity of culturable denitrifying community between the overlying water and other two phases (biofilm and sediment) (Fig. 8). Specifically, the OTU 2 (*Pm. stutzeri*) accounted for 37.8% of the culturable aerobic denitrifiers in overlying water but only had the percentage of 12.2% and 6.7% in the phases of biofilm and sediment respectively (Fig. 6). The OTU 7 (*O. rhizosphaerae*) was the fourth abundant culturable aerobic denitrifier in water which occupied 6.7% of the relative abundance. Nonetheless, the strains of OTU 7 (*O. rhizosphaerae*) were not isolated from the biofilm and sediment samples.

## Discussion

In order to purify the nitrate contaminated river, it is necessary to isolate the efficient denitrifying bacteria which can adapt to different niches. In this study, three species of the genus *Pseudomonas*, including the *Pm. stutzeri*, *Pm. mendocina* and *Pm. putida*, were found to be the common abundant aerobic denitrifying bacteria in the phases of overlying water, biofilm and sediment, indicating they may be the universal dominant culturable aerobic denitrifiers. The conclusion here was supported by the previous studies, where the *Pm. stutzeri*, *Pm. mendocina* and *Pm. putida* were all found to have the denitrifying ability under different aerobic conditions^15, 18, 25^. For example, the *Pm. stutzeri* had been found to distribute widely in the environment, such as the wastewater and sediment of fishponds^16, 26^, soil of silver mine^27^, groundwater, marine water and wastewater treatment plants^28^. Furthermore, a strain of *Pseudomonas stutzeri* isolated from the wastewater treatment plants, *Pm. stutzeri* TR2, was also reported to promote the transformation of N_2_O to N_2_ and therefore decrease the emission of N_2_O^15^, which is a significant contributor to the global warming^29^. Thus, the dominant existence of the *Pm. stutzeri* in the culturable aerobic denitrifying community in this study indicated that the *Pm. stutzeri* may be used in the nitrate removal from the surface water and play an important role in the prevention of global warming. The *Pm. putida* was another denitrifying species that had been proven to tolerate 5.0–6.0 mg/L of the oxygen^18^. The detection of nitrate reductase from the periplasm of *Pm. putida* was a further demonstration of the denitrifying ability for this bacterium^30^. The *Pm. monteilii* was reported to be highly similar to the *Pm. putida* based on the 16S rDNA and can only be differentiated by the different substrate utilization of inositol, α-aminobutyrate, and o-/m-hydroxybenzoate^31^. This conclusion was also supported by this study, where the dissimilarity of the 16S rDNA was below 1% between the *Pm. putida* and *Pm*. monteilii (Figs 3–5). Further, the data here showed another common character of the *Pm. monteilii* and *Pm. putida*, which is the nitrate reducing property.

In addition to the dominant *Pseudomonas* species, bacteria of other genera also presented the denitrifying ability in the presence of oxygen gas, such as the *Ochrobactrum*, *Pannonibacter* (*Pn*), *Gordonia* and *Stenotrophomonas*. The nitrate reduction ability of *O. tritici* and *O. anthropi* was also reported in a previous study^32^. Interestingly, the *Pn. Phragmitetus* had been proven to perform denitrification under anaerobic condition^33^, but this study indicated that this species was also capable of reducing the nitrate in the presence of oxygen gas. For the genus of *Gordonia* sp., it was known that one species of *G. sihwensis* was aerobic nitrate reducing bacterium^34^. However, few reports showed the *G. terrae* and *G. malaquae* were both capable of aerobic denitrification. So this study is the first report to show the aerobic denitrifying ability of *G. terrae* and *G. malaquae*. Two species of *Stenotrophomonas*, including *S. terrae* and *S. acidaminiphila*, were isolated from the overlying water in this study. The nitrate reducing character of *S. terrae* had already been reported^35^, while the *S. acidaminiphila* was first reported to be an aerobic denitrifier in this study. Some other bacteria were also first reported to have denitrifying ability in this study, including the *Pc. Saccharolyticum*, *O. rhizosphaerae*, *A. ureafaciens* and *Rh. Pacifica*. An important phenomenon needs to be noticed that some aerobic denitrifying bacteria were reported to often have the ammonia oxidizing ability, such as *Pm. stutzeri* ^26^, *Pm. putida* ^36^, *Paracoccus denitrificans* ^37^ and *Klebsiella pneumoniae* ^38^. This facilitates the simultaneously aerobic nitrification and denitrification processes, which are different from the traditional nitrogen removal processes through the aerobic nitrification and anaerobic denitrification. The strains of *Pm. stutzeri* and *Pm. putida* were also isolated in this study. However, the simultaneously nitrifying and denitrifying ability of these isolates have not been tested. Therefore, a task in the future work may be to determine the ammonia oxidizing capability of these isolated aerobic denitrifying bacteria.

The isolation of the aerobic denitrifying bacteria is a premise for the bioaugmentation-based water purification. Bioaugmentation is a strategy to promote the degradation of contaminants through introducing the highly efficient pollutant-degrading bacteria to the environment or wastewater treatment plant^39^. For the *in situ* remediation of polluted river through bioaugmentation, two environmental sites including the sediment and overlying water are usually selected. The site selection determines the way of bioaugmentation, e.g. depositing the pollutant-degrading bacteria immobilized gel beads into the sediment or pouring the solution of pollutant-degrading bacteria into the contaminated water directly^40^. In addition, it was known that some bacteria in aquatic phase can attached onto certain surface to form the biofilms^41^. Biofilm is a microbial sessile living mode where the bacteria aggregate together onto the surface to develop a three-dimensional structure and were regulated by the quorum sensing mechanisms^41^. In the biofilm structure, the bacteria usually owned some different characters which are different from the planktonic living bacteria in water, e.g. secretion of extracellular polysaccharides^41^. The formation of biofilms may increase the biomass and duration of bacteria in water. Thus it was also regarded as a bioaugmentation method by introducing the biofilm bed to the water phase^42^. In combination, the selection of environmental sites and bioaugmentation modes determine the types of bacteria which would be used to conduct the purification function in surface water. The unevenly distribution of richness shown by the Venn diagram (Fig. 2) indicated that the structure of aerobic denitrifiers may be different between the phases of biofilm, sediment and water. Examined by the cluster analysis of the 16S rDNA, the culturable aerobic denitrifying communities had only 38.1% in Bray-Curtis similarity among the three phases of Liangshui River (Fig. 6), which was a confirmation that the culturable aerobic denitrifiers had different community structures in the three phases of river. Moreover, the aerobic denitrifying community in biofilm and sediment had a closer relationship (55.2% in Bray-Curtis similarity) relative to the aerobic denitrifying bacteria in water. This is consistent with the data of richness distribution, implying the community of culturable aerobic denitrifiers had more similar community compositions in the biofilm and sediment phases, in comparison with the planktonic living phase in overlying water. It is known that the bacteria own a sessile living mode in the biofilm, where a surface is needed for the attachment of bacteria. This is the reason why we provided a polypropylene supporting material for the growth of biofilm (Supplementary Fig. S6). The sediment is usually composed of microorganisms, humic substances, soil, sands, etc. So the soil and sands in sediment may offer the surface for the bacteria to form biofilm structures. This may explain why the culturable denitrifiers in the biofilms on polypropylene supporting material had more similar community structure to the sediment rather than the overlying water.

In biofilm phase, the OTU 1 (*Pm. mendocina*) had the highest relative abundance, implying the *Pm. mendocina* may have the property to inhabit in biofilm. This is consistent to the previous studies, in which the *Pm. mendocina* was demonstrated to be the initial colonizers of biofilm and promoted the growth of biofilm aggregations through the production of cell bounded extracellular polysaccharides^43^. For the OTU 5 (*Rh. pacifica*) it was only isolated and enriched in the biofilm samples. This may indicate the *Rh. pacifica* was the rare organisms in water and sediment but had strong biofilm forming ability, leading to the thriving and enrichment of the OTU in biofilms. These findings also implied that the *Pm. mendocina* and *Rh. pacifica* may be suitable to be used in the biofilm-based bioaugmentation. Similarly, the OTU 6 (*Pseudomonas resinovorans*) and OTU 14 (*Brevundimonas diminuta*) may be better to be introduced into the sediment, while the OTU 2 (*Pm. stutzeri*) and OTU 7 (*O. rhizosphaerae*) may give a better growth in the overlying water. It was also noted that some OTUs can only be detected in one phase, such as the OTUs 6, 12, 14 and 18 isolated from sediment, the OTUs 7, 11, 17, 19 and 21 isolated from overlying water and the OTUs 5, 22, 23 and 24 isolated from biofilm. However, it may be hard to judge that there were no such bacteria in the other two phases of Liangshui River, as the abundance of these microorganisms may be quite low which increased the difficulty to isolate them. Therefore, the knowledge here is helpful to select the appropriate niche in the isolation of aerobic denitrifying bacteria and may increase the possibility to get the targeted bacteria.

In conclusion, a total of 116 strains of aerobic denitrifying bacteria were isolated from the sediment, overlying water and biofilms on supporting materials in Liangshui River of Beijing, which were classified into 14 genera based on the 16S rDNA, such as *Pseudomonas*, *Rheinheimera*, and *Pannonibacter*. Three species of *Pseudomonas*, including the *Pm. stutzeri*, *Pm. mendocina* and *Pm. putida*, were the dominant and universal culturable aerobic denitrifying bacteria in all the phases of river. These culturable aerobic denitrifiers had different distribution characteristics among the 3 phases of Liangshui River. The isolation of aerobic denitrifiers would facilitate the purification of the nitrate polluted surface water, e.g. contaminated river and lake. However, the optimal working conditions of these denitrifiers, such as pH, ratio of carbon to nitrogen (C/N) and removal efficiency, had not been revealed. Therefore, the future works may focus on the determination and optimization of removal efficiencies and working conditions. Furthermore, the screening of the ammonia oxidizing ability also needs to be done for these isolated aerobic denitrifiers, in order to find the simultaneous nitrifying and aerobic denitrifying bacteria.

## Methods

### Sample collection and measurement of water quality

The sampling points were selected at 500 meters (m) downstream of the outfall point of Xiaohongmen wastewater treatment plant, which located near the Liangshui River of Beijing and drain off the treated water to Liangshui River (Fig. S1). Three types of samples, which included the sediment, overlying water and biofilms, were collected in Liangshui River as the microbial reservoir to isolate the aerobic denitrifying bacteria. Samples of Liangshui River were collected in March 2015 at three niches: overlying water (at 50 cm depth), sediment (at 100 cm depth) and biofilm (at 50 cm depth). In order to isolate aerobic denitrifying bacteria from the biofilm samples, pieces of polypropylene supporting material had been immersed and suspended in the Liangshui River for 6 months (Supplementary Fig. S6). The water quality in the Liangshui River of Beijing in China was determined based on the contamination of ammonia and nitrate. The concentration of ammonia in the water was measured based on the Nessler’s reaction^44^. The concentration of nitrate was quantified by the thymol solution^45^.

### Media, isolation and screening of the aerobic denitrifying bacteria

Three types of media were used in the isolation and screening of the aerobic denitrifying bacteria, including the enrichment medium (EM), denitrifying medium (DM), and screen medium (GN). The EM was composed of 0.5 g/L (NH_4_)_2_SO_4_, 0.36 g/L KNO_3_, 4.0 g/L sodium citrate, 0.05% (ratio of volume) of the trace element solution which was composed of 6.5 g/L K_2_HPO_4_·3H_2_O, 2.5 g/L MgSO_4_·7H_2_O, 2.5 g/L NaCl, 0.05 g/L FeSO_4_·7H_2_O, 0.04 g/L MnSO_4_·H_2_O. The final pH of the EM was adjusted to 7.0. The DM was composed of 0.36 g/L KNO_3_, 10.55 g/L Na_2_HPO_4_·12H_2_O, 1.5 g/L KH_2_PO_4_, 0.1 g/L MgSO_4_·7H_2_O, 4.0 g/L sodium citrate, 0.2% (volume ratio) of trace element solution which included 50.0 g/L EDTA-Na_2_, 2.2 g/L ZnSO_4_, 5.5 g/L CaCl_2_, 5.06 g/L MnCl_2_·4H_2_O, 5.0 g/L FeSO_4_·7H_2_O, 1.57 g/L CuSO_4_·5H_2_O, 1.61 g/L CoCl_2_·6H_2_O. The final pH of the DM was adjusted to 7.0. The GN for the denitrifying bacteria was composed of 1.0 g/L KNO_3_, 8.5 g/L sodium citrate, 1.0 g/L L-asparagine, 1.0 g/L KH_2_PO_4_, 1.0 g/L MgSO_4_·7H_2_O, 0.2 g/L CaCl_2_·6H_2_O, 0.05 g/L FeCl_3_·6H_2_O, 0.1% (volume ratio) of the 1% (ratio of weight/volume) alcoholic dissolved bromothymol blue (BTB)^46^. The final pH of the DM was adjusted to 7.0. The solid GN was made by adding 2% of agar powder to the liquid GN.

The isolation and screening of denitrifying bacteria were based on the production of hydroxide ion (OH^−^) due to the reduction of nitrate in denitrification process. A complete denitrifying process is composed of 4 steps which were shown as the Eqs (1–4):

1 $$8{{\rm{NO}}}_{3}^{-}+2{{\rm{CH}}}_{3}{\rm{COOH}}\to 8{{\rm{NO}}}_{2}^{-}+4{{\rm{CO}}}_{2}+4{{\rm{H}}}_{2}{\rm{O}}$$

2 $$8{{\rm{NO}}}_{2}^{-}+{{\rm{CH}}}_{3}{\rm{COOH}}+2{{\rm{H}}}_{2}{\rm{O}}\to 8{\rm{NO}}+2{{\rm{CO}}}_{2}+8{{\rm{OH}}}^{-}$$

3 $$8{\rm{NO}}+{{\rm{CH}}}_{3}{\rm{COOH}}\to 2{{\rm{CO}}}_{2}+4{{\rm{N}}}_{2}{\rm{O}}+2{{\rm{H}}}_{2}{\rm{O}}$$

4 $$4{{\rm{N}}}_{2}{\rm{O}}+{{\rm{CH}}}_{3}{\rm{COOH}}\to 2{{\rm{CO}}}_{2}+4{{\rm{N}}}_{2}+2{{\rm{H}}}_{2}{\rm{O}}$$

Overall, the denitrifying process can be displayed as the Eq. (5)

5 $$8{{\rm{NO}}}_{3}^{-}+5{{\rm{CH}}}_{3}{\rm{COOH}}\to 4{{\rm{N}}}_{2}+10{{\rm{CO}}}_{2}+6{{\rm{H}}}_{2}{\rm{O}}+8{{\rm{OH}}}^{-}$$

In the equations, the acetate (CH_3_COOH) is used as the carbon source and electron donor for the denitrification and the OH^−^ is produced as a byproduct of the reduction of nitrite to nitric oxide, which will increase the pH of the reaction system^47^. The GN in this study was composed of nutrient elements and the pH indicating dye BTB, which would change the color from green to blue when the pH increases from 7.0 to high levels. If the bacteria growing on the solid GN (pH 7.0) have the denitrifying ability, the pH in the medium surrounding the bacterial colonies would increase, resulting in the color of the colonies turns to the blue^19^.

Samples from the phases of water, biofilm and sediment were transferred and inoculated into 250 mL EM and incubated at 30 °C with shaking at 150 r/min for 30 days (d). During the enrichment stage, 25 mL of mixed solution of EM and microorganisms were sucked out and replaced with the fresh EM every 48 hours (hr). From the d 31, 25 mL of the mixed solution of medium and microorganisms were sucked out and replaced with the fresh DM every 24 hr. After the incubation with DM for 14 d, the isolation and screening of the aerobic denitrifying bacteria was conducted. For the samples of water phase, 1 mL of the mixed cultures were diluted and spread on solid GN. For the isolation from the biofilm phase, 30 mL of the mixed solutions of DM and biofilm samples were transferred to sterile 50 mL tubes which contained sterile glass beads. The tubes containing biofilm samples were shaken at 5000 r/min on a vortex mixer (VORTEX-GENIE 2) to detach the bacteria from the biofilms. Next, 1 mL of mixed solution of DM and detached bacteria were diluted to spread on the solid GN. The procedure of bacteria isolation from sediment was similar to the biofilm samples, where the biofilm samples were replaced by the sediment. The solid GN plates were incubated at 30 °C. During the incubation, the blue colonies were separately picked out and incubated in liquid GN at 30 °C with shaking at 150 r/min to confirm the change of color (Supplementary Fig. S6).

### Measurement and re-confirmation of aerobic denitrifying ability

24 isolates were selected as the representative strains of 24 OTUs (Table 1). For nitrate removal, a 10 mL bacteria suspension under logarithmic phase was centrifuged at 5000 rpm for 5 mins and then rinsed with 1 × PBS (phosphate buffered saline) for 3 times to remove the excess medium. Resuspended the bacteria with 1 × PBS, inoculated into 1 mL denitrifying medium (DM), and incubated at 150 rpm at 30 °C for 96 h. Each treatment was carried out in triplicate. The aerobic denitrification efficiency (i.e., the TN removal efficiency) were calculated by the Eq. (6):

6 $$\mathrm{TN\; removal\; efficiency}=\frac{\mathrm{CK}(\mathrm{TN})-\mathrm{EG}(\mathrm{TN})}{\mathrm{CK}(\mathrm{TN})}\times 100 \% $$

In the equation, the CK (TN) represents the initial total nitrogen of the treatments, the EG (TN) is the terminal total nitrogen after 96 h. Concentration of TN was determined by the method of Alkaline potassium persulfate digestion-uv spectrophotometer^48^.

### Colony PCR and sequencing

The 16S rDNA sequences for the isolated aerobic denitrification bacteria were amplified through a colony PCR procedure^49^. The primers for the 16S rDNA were composed of 27 F (5′-AGAGTTTGATCMTGGCTCAG-3′) and 1492R (5′-TACGGYTACCTTGTTACGACTT-3′)^18^. The PCR procedure was composed of a pre-denaturing step at 95 °C for 5 min followed by 35 cycles of denaturing at 95 °C for 30 s, annealing at 55 °C for 45 s and extending at 72 °C for 1.5 min and 1 cycle at 72 °C for 10 min. The PCR products were sequenced by the Sanger sequencing method. All the sequences had been deposit in the GenBank. The accession numbers are KT380544 – KT380588 for the isolates from the overlying water (Supplementary Table S2), KT380503 – KT380543 for the bacteria isolated from the biofilm samples (Supplementary Table S3) and KT380589 – KT380619 for the bacteria isolated from the sediment of the Liangshui River (Supplementary Table S4).

### Sequence classification and phylogenetic analysis

All sequences acquired from the Sanger sequencing method were classified to the taxonomic ranks against to the RDP 16S rRNA training set 14 and NCBI 16S ribosome RNA databases respectively^50^. The information of the taxonomic ranks from Kingdom to genus was generated by the RDP classification at the confidence threshold of 95%. The closest species and strains were picked from the NCBI blasting results based on the highest score, sequence coverage and identity. The bootstrap tested neighbor joining phylogenetic trees were constructed in MEGA 4.0 for the sequences of isolated strains and reference strains. The bootstrap value for the sequence classification was set to be 60.

### OTU assignment and community analysis

An OTU table based on the 16S rDNA was formed for the sequences of all isolated aerobic denitrifiers using the MOTHUR software. First, three different barcode sequences were added to the sequences of the water, sediment and biofilm communities separately to distinguish amplicon sequences from different communities. Next, the sequences of the three groups were merged together by the command “merge.files” and re-assigned to different groups by the process of “trim.seqs”^51, 52^, in which the barcode sequences were removed at the same time. The sequences were then aligned to the Silva bacterial reference data by the “align.seqs” and the nonsense columns which only contained the character of “.” were removed by the process of “filter.seqs”. Finally, after the calculation of the distances using the command “dist.seqs”, the sequences were clustered and assigned to OTUs at the cutoff of 0.03 through the processes of “cluster” and “make.shared”^51, 52^. The OTU-based similarity or dissimilarity between the culturable communities in 3 phases was calculated by “SIMPER” analysis in PRIMER 6.0 (PRIMER-E). The contributions of the OTUs to the similarity or dissimilarity were calculated based on the relative abundances of OTUs between the groups. Clustering trees and principal component analysis (PCA) plot were created between the samples based on the average Bray-Curtis similarity of OTUs compositions in the different groups of the overlying water, sediment and biofilm^52^.

## Electronic supplementary material

Supplementary information
