# Supplementary material for: Diversity of culturable aerobic denitrifying bacteria in the sediment, water and biofilms in Liangshui River of Beijing, China
Source: Sci Rep. 2017 Aug 30;7:10032. doi: 10.1038/s41598-017-09556-9 (PMC5577267; doi:10.1038/s41598-017-09556-9)
Supplement: Supplementary file 1 — Supplementary information [file 41598_2017_9556_MOESM1_ESM.pdf]

# Supplementary information

**Diversity of culturable aerobic denitrifying bacteria in the sediment, water and biofilms in Liangshui River of Beijing, China.**

Pengyi Lv[1,2,†], Jinxue Luo[2,†], Xuliang Zhuang[2,3,*], Dongqing Zhang[4], Zhanbin Huang[1,*] & Zhihui Bai[2, 3,*]

[1]School of Chemical and Environmental Engineering, China University of Mining and Technology (Beijing), Beijing 100083, China. [2]Research Center for Eco-Environmental Sciences, Chinese Academy of Sciences, Beijing 100085, China. [3]College of Resources and Environment, University of Chinese Academy of Sciences, Beijing 100049, China. [4]Advanced Environmental Biotechnology Centre, Nanyang Environment and Water Research Institute, Nanyang Technological University, Singapore 637141.

† These authors contributed equally to this work.

*Corresponding authors. E-mail: zhbai@rcees.ac.cn (Zhihui Bai), xlzhuang@rcees.ac.cn (Xulinag Zhuang) or zbhuang2003@163.com (Zhanbin Huang)

**Table S1. The aerobic denitrifying bacteria in each OTU at cutoff of 0.03.**

| OTUs | Strains of aerobic denitrifying bacteria |
|---|---|
| OTU 1 | W6, W16, W19, W20, W22, W26, W29, W33, W35, B13, B17, B19, B20, B21, B22, B25, B26, B27, B43, B44, S18, S25, S27,S32 |
| OTU 2 | W1, W11, W12, W13, W15, W18, W21, W25, W28, W31, W32, W34, W36,W41, W42, W43, W45, B1, B11, B15, B36, B41, S1, S2 |
| OTU 3 | W7, W17, W23, W27, W37, W44, B2, B4, B10, B30, B31, B45, S10, S15, S22, S23, S28, S29, S30, S33 |
| OTU 4 | B12, S4, S5, S13, S14, S16 |
| OTU 5 | B24, B32, B33, B39 |
| OTU 6 | S7, S9, S11, S12 |
| OTU 7 | W24, W40, W46 |
| OTU 8 | W30, W38, B16 |
| OTU 9 | B14, B18, B23, B28, B29, B34, B35, B40, B42, S24, S26, S31 |
| OTU 10 | B38, S19 |
| OTU 11 | W9 |
| OTU 12 | S3 |
| OTU 13 | W4 |
| OTU 14 | S20 |
| OTU 15 | W3 |
| OTU 16 | W5 |
| OTU 17 | W2 |
| OTU 18 | S6 |
| OTU 19 | W14 |
| OTU 20 | W8 |
| OTU 21 | W10 |
| OTU 22 | B8 |
| OTU 23 | B7 |
| OTU 24 | B5 |

**Table S2. The aerobic denitrifying bacteria in overlying water and their closest relative reference strains in NCBI database.**

| Strain Number | Related GenBank sequence | Closest relative (NCBI accession No.) | Identity (%) |
|---|---|---|---|
| W1 | KT380544 | *Pseudomonas stutzeri* A1501(NR074829) | 99.79 |
| W3 | KT380545 | *Gordonia terrae* strain 3612(NR037022) | 99.50 |
| W4 | KT380546 | *Stenotrophomonas acidaminiphila* strain AMX 19 (NR025104) | 99.43 |
| W9 | KT380547 | *Gordonia malaquae* strain IMMIB WWCC-22 (NR115020) | 98.72 |
| W27 | KT380548 | *Pseudomonas putida* F1(NR074739) | 99.72 |
| W37 | KT380549 | *Pseudomonas monteilii*(NR121767) | 99.65 |
| W43 | KT380550 | *Pseudomonas stutzeri* A1501(NR074829) | 99.72 |
| W2 | KT380551 | *Comamonas terrigena* strain NBRC 12685 (NR113597) | 96.96 |
| W5 | KT380552 | *Paracoccus versutus* strain NBRC 14567 (NR113662) | 99.55 |
| W6 | KT380553 | *Pseudomonas mendocina* ymp(NR074727) | 99.79 |
| W7 | KT380554 | *Pseudomonas monteilii*(NR121767) | 99.93 |
| W8 | KT380555 | *Stenotrophomonas terrae* strain R-32768 | 98.68 |
| W10 | KT380556 | *Arthrobacter ureafaciens* strain NC(NR029281) | 98.8 |
| W11 | KT380557 | *Pseudomonas stutzeri* A1501(NR074829) | 99.72 |
| W12 | KT380558 | *Pseudomonas stutzeri* A1501(NR074829) | 99.93 |
| W13 | KT380559 | *Pseudomonas stutzeri* A1501(NR074829) | 99.72 |
| W14 | KT380560 | *Pseudochrobactrum saccharolyticum* strain CCUG 33852(NR042473) | 99.93 |
| W15 | KT380561 | *Pseudomonas stutzeri* A1501(NR074829) | 99.65 |
| W16 | KT380562 | *Pseudomonas mendocina* ymp(NR074727) | 99.79 |
| W17 | KT380563 | *Pseudomonas putida* KT2440(NR074596) | 99.65 |
| W18 | KT380564 | *Pseudomonas stutzeri* A1501(NR074829) | 99.79 |
| W19 | KT380565 | *Pseudomonas mendocina* ymp(NR074727) | 99.72 |
| W20 | KT380566 | *Pseudomonas mendocina* strain ATCC 25411 (NR114477) | 98.94 |
| W21 | KT380567 | *Pseudomonas stutzeri* A1501(NR074829) | 99.44 |
| W22 | KT380568 | *Pseudomonas mendocina* strain ATCC 25411 (NR114477) | 98.74 |
| W23 | KT380569 | *Pseudomonas monteilii*(NR121767) | 99.72 |
| W24 | KT380570 | *Ochrobactrum rhizosphaerae* strain PR17 (NR042600) | 99.27 |
| W25 | KT380571 | *Pseudomonas stutzeri* A1501(NR074829) | 99.86 |

| | | | |
|---|---|---|---|
| W26 | KT380572 | *Pseudomonas mendocina* strain ATCC 25411 (NR114477) | 98.67 |
| W28 | KT380573 | *Pseudomonas stutzeri* A1501(NR074829) | 99.51 |
| W29 | KT380574 | *Pseudomonas mendocina* ymp (NR074727) | 99.50 |
| W30 | KT380575 | *Pannonibacter phragmitetus* strain C6-19 (NR028009) | 99.35 |
| W31 | KT380576 | *Pseudomonas stutzeri* A1501(NR074829) | 99.79 |
| W32 | KT380577 | *Pseudomonas stutzeri* A1501(NR074829) | 99.86 |
| W33 | KT380578 | *Pseudomonas mendocina* ymp(NR074727) | 99.65 |
| W34 | KT380579 | *Pseudomonas stutzeri* A1501(NR074829) | 99.72 |
| W35 | KT380580 | *Pseudomonas mendocina* ymp(NR074727) | 99.72 |
| W36 | KT380581 | *Pseudomonas stutzeri* A1501(NR074829) | 99.72 |
| W38 | KT380582 | *Pannonibacter phragmitetus* strain C6-19 (NR028009) | 99.19 |
| W40 | KT380583 | *Ochrobactrum rhizosphaerae* strain PR17 (NR042600) | 98.75 |
| W41 | KT380584 | *Pseudomonas stutzeri* A1501(NR074829) | 99.79 |
| W42 | KT380585 | *Pseudomonas stutzeri* A1501(NR074829) | 99.65 |
| W44 | KT380586 | *Pseudomonas putida* KT2440 (NR074596) | 99.72 |
| W45 | KT380587 | *Pseudomonas stutzeri* A1501(NR074829) | 99.86 |
| W46 | KT380588 | *Ochrobactrum rhizosphaerae* strain PR17 (NR042600) | 99.19 |

**Table S3. The aerobic denitrifying bacteria in biofilm phase and their closest relative reference strains in NCBI database.**

| Strain Number | Related GenBank sequence | Closest relative (NCBI accession No.) | Identity (%) |
|---|---|---|---|
| B4 | KT380503 | *Pseudomonas putida* F1 (NR074739) | 99.64 |
| B5 | KT380504 | *Arthrobacter soli* strain SYB2(NR044338) | 99.86 |
| B7 | KT380505 | *Rhodococcus pyridinivorans* strain DSM 44555 (NR118620) | 99.93 |
| B8 | KT380506 | *Gemmobacter caeni* strain DCA-1(NR108321) | 99.85 |
| B16 | KT380507 | *Pannonibacter phragmitetus* strain C6-19 (NR028009) | 99.27 |
| B30 | KT380508 | *Pseudomonas putida* KT2440 (NR074596) | 99.37 |
| B36 | KT380509 | *Pseudomonas stutzeri* A1501(NR074829) | 99.93 |
| B38 | KT380510 | *Rhizobium pusense* strain NRCPB10(NR116874) | 99.27 |
| B44 | KT380511 | *Pseudomonas mendocina* ymp(NR074727) | 98.73 |
| B45 | KT380512 | *Pseudomonas monteilii* (NR121767) | 99.86 |
| B1 | KT380513 | *Pseudomonas stutzeri* A1501 (NR074829) | 99.79 |
| B2 | KT380514 | *Pseudomonas putida* KT2440(NR074596) | 99.79 |
| B10 | KT380515 | *Pseudomonas putida* KT2440(NR074596) | 99.86 |
| B11 | KT380516 | *Pseudomonas stutzeri* A1501(NR074829) | 99.86 |
| B12* | KT380517 | *Ochrobactrum anthropi* strain ATCC 49188 (NR074243) | 99.85 |
| | | *Ochrobactrum cytisi* strain ESC1(NR043184) | 99.85 |
| | | *Ochrobactrum lupini* strain LUP21(NR042911) | 99.85 |
| | | *Ochrobactrum tritici* strain SCII24(NR028902) | 99.85 |
| B13 | KT380518 | *Pseudomonas mendocina* ymp(NR074727) | 99.65 |
| B14* | KT380519 | *Pseudomonas chengduensis* strain MBR (NR125523) | 99.58 |
| | | *Pseudomonas toyotomiensis* strain HT-3 (NR112808) | 99.58 |
| | | *Pseudomonas oleovorans* RS1(NR115874) | 99.58 |
| B15 | KT380520 | *Pseudomonas stutzeri* A1501(NR074829) | 99.65 |
| B17 | KT380521 | *Pseudomonas mendocina* ymp(NR074727) | 99.44 |
| B18* | KT380522 | *Pseudomonas chengduensis* strain MBR (NR125523) | 98.12 |
| | | *Pseudomonas oleovorans* RS1(NR115874) | 98.32 |
| B19 | KT380523 | *Pseudomonas mendocina* ymp(NR074727) | 99.72 |
| B20 | KT380524 | *Pseudomonas mendocina* ymp(NR074727) | 99.72 |

| | | | |
|---|---|---|---|
| B21 | KT380525 | *Pseudomonas mendocina* ymp(NR074727) | 98.53 |
| B22 | KT380526 | *Pseudomonas mendocina* ymp(NR074727) | 98.74 |
| B23* | KT380527 | *Pseudomonas chengduensis* strain MBR (NR125523) | 98.05 |
| | | *Pseudomonas oleovorans* RS1(NR115874) | 98.11 |
| B24 | KT380528 | *Rheinheimera pacifica* strain NBRC 103167 | 97.88 |
| B25 | KT380529 | *Pseudomonas mendocina* ymp(NR074727) | 99.51 |
| B26 | KT380530 | *Pseudomonas mendocina* ymp(NR074727) | 99.44 |
| B27 | KT380531 | *Pseudomonas mendocina* ymp(NR074727) | 99.37 |
| B28* | KT380532 | *Pseudomonas toyotomiensis* strain HT-3 (NR112808) | 99.58 |
| | | *Pseudomonas chengduensis* strain MBR (NR125523) | 99.58 |
| B29* | KT380533 | *Pseudomonas toyotomiensis* strain HT-3 (NR112808) | 99.43 |
| | | *Pseudomonas chengduensis* strain MBR (NR125523) | 99.43 |
| B31 | KT380534 | *Pseudomonas putida* F1(NR074739) | 99.51 |
| B32 | KT380535 | *Rheinheimera pacifica* strain NBRC 103167 (NR114230) | 97.73 |
| B33 | KT380536 | *Rheinheimera pacifica* strain NBRC 103167 (NR114230) | 97.54 |
| B34* | KT380537 | *Pseudomonas toyotomiensis* strain HT-3 (NR112808) | 99.51 |
| | | *Pseudomonas chengduensis* strain MBR (NR125523) | 99.51 |
| | | *Pseudomonas oleovorans* RS1(NR115874) | 99.51 |
| B35* | KT380538 | *Pseudomonas toyotomiensis* strain HT-3 (NR112808) | 99.65 |
| | | *Pseudomonas chengduensis* strain MBR (NR125523) | 99.65 |
| | | *Pseudomonas oleovorans* RS1(NR115874) | 99.65 |
| B39 | KT380539 | *Rheinheimera pacifica* strain NBRC 103167 (NR114230) | 97.66 |
| B40* | KT380540 | *Pseudomonas chengduensis* strain MBR (NR125523) | 99.44 |
| | | *Pseudomonas toyotomiensis* strain HT-3 (NR112808) | 99.44 |
| B41 | KT380541 | *Pseudomonas stutzeri* A1501(NR074829) | 99.79 |
| B42* | KT380542 | *Pseudomonas oleovorans* RS1(NR115874) | 98.44 |
| | | *Pseudomonas chengduensis* strain MBR (NR125523) | 98.30 |
| B43 | KT380543 | *Pseudomonas mendocina* ymp(NR074727) | 99.23 |

*means the isolated bacterium was unclassified at Species level based on the 16S rRNA sequence.

**Table S4. The aerobic denitrifying bacteria in sediment phase and their closest relative reference strains in NCBI database.**

| Strain Number | Related GenBank | Closest relative (NCBI accession No.) | Identity (%) |
|---|---|---|---|
| S6 | KT380589 | *Rhodococcus canchipurensis* strain MBRL 353 (NR109454) | 99.07 |
| S14* | KT380590 | *Ochrobactrum tritici* SCII24(NR114980) | 99.78 |
| | | *Ochrobactrum anthropi* strain ATCC 49188 | 99.49 |
| S20 | KT380591 | *Brevundimonas diminuta* strain NBRC 12697 (NR113602) | 99.78 |
| S1 | KT380592 | *Pseudomonas stutzeri* A1501 (NR074829) | 99.50 |
| S2 | KT380593 | *Pseudomonas stutzeri* A1501 (NR074829) | 99.30 |
| S3 | KT380594 | *Gordonia alkanivorans* strain HKI 0136 (NR026488) | 99.50 |
| S4* | KT380595 | *Ochrobactrum anthropi* strain ATCC 49188 (NR074243) | 99.64 |
| | | *Ochrobactrum cytisi* strain ESC1 (NR043184) | 99.64 |
| S5* | KT380596 | *Ochrobactrum tritici* strain NBRC 102585 (NR114148) | 99.63 |
| | | *Ochrobactrum anthropi* strain ATCC 49188 (NR074243) | 99.34 |
| S7 | KT380597 | *Pseudomonas resinovorans* NBRC 106553 (NR103921) | 98.80 |
| S9 | KT380599 | *Pseudomonas resinovorans* NBRC 106553 (NR103921) | 98.31 |
| S10 | KT380600 | *Pseudomonas putida* KT2440 (NR074596) | 99.65 |
| S11 | KT380601 | *Pseudomonas resinovorans* NBRC 106553 (NR103921) | 98.80 |
| S12 | KT380602 | Pseudomonas resinovorans NBRC 106553 (NR103921) | 98.93 |
| S13* | KT380603 | *Ochrobactrum tritici* SCII24(NR114980) | 99.56 |
| | | *Ochrobactrum anthropi* strain ATCC 49188(NR074243) | 99.27 |
| S15 | KT380604 | *Pseudomonas putida* KT2440(NR074596) | 99.72 |
| S16* | KT380605 | *Ochrobactrum tritici* SCII24(NR114980) | 99.41 |
| | | *Ochrobactrum anthropi* strain ATCC 49188(NR074243) | 99.12 |
| S18 | KT380606 | *Pseudomonas mendocina* ymp(NR074727) | 99.58 |
| S19 | KT380607 | *Rhizobium pusense* strain NRCPB10 (NR116874) | 99.50 |
| S22 | KT380608 | *Pseudomonas putida* F1(NR074739) | 99.51 |
| S23 | KT380609 | *Pseudomonas putida* KT2440 (NR074596) | 99.65 |
| S24* | KT380610 | *Pseudomonas toyotomiensis* strain HT-3 (NR112808) | 99.51 |
| | | *Pseudomonas chengduensis* strain MBR (NR125523) | 99.51 |

| | | | |
|---|---|---|---|
| S25 | KT380611 | *Pseudomonas mendocina* ymp(NR074727) | 99.71 |
| S26* | KT380612 | *Pseudomonas chengduensis* strain MBR (NR125523) | 99.58 |
| | | *Pseudomonas toyotomiensis* strain HT-3 (NR112808 ) | 99.58 |
| S27 | KT380613 | *Pseudomonas mendocina* ymp (NR074727) | 99.51 |
| S28 | KT380614 | *Pseudomonas putida* KT2440(NR074596) | 99.37 |
| S29 | KT380615 | *Pseudomonas monteilii* (NR121767) | 99.79 |
| S30 | KT380616 | *Pseudomonas monteilii* (NR121767) | 99.79 |
| S31* | KT380617 | *Pseudomonas toyotomiensis* strain HT-3 (NR112808) | 99.65 |
| | | *Pseudomonas chengduensis* strain MBR (NR125523) | 99.65 |
| S32 | KT380618 | *Pseudomonas mendocina* ymp(NR074727) | 98.74 |
| S33 * | KT380619 | *Pseudomonas monteilii* (NR121767) | 99.37 |
| | | *Pseudomonas putida* F1(NR074739) | 99.23 |

*means the isolated bacterium was unclassified at Species level based on the 16S rRNA sequence.

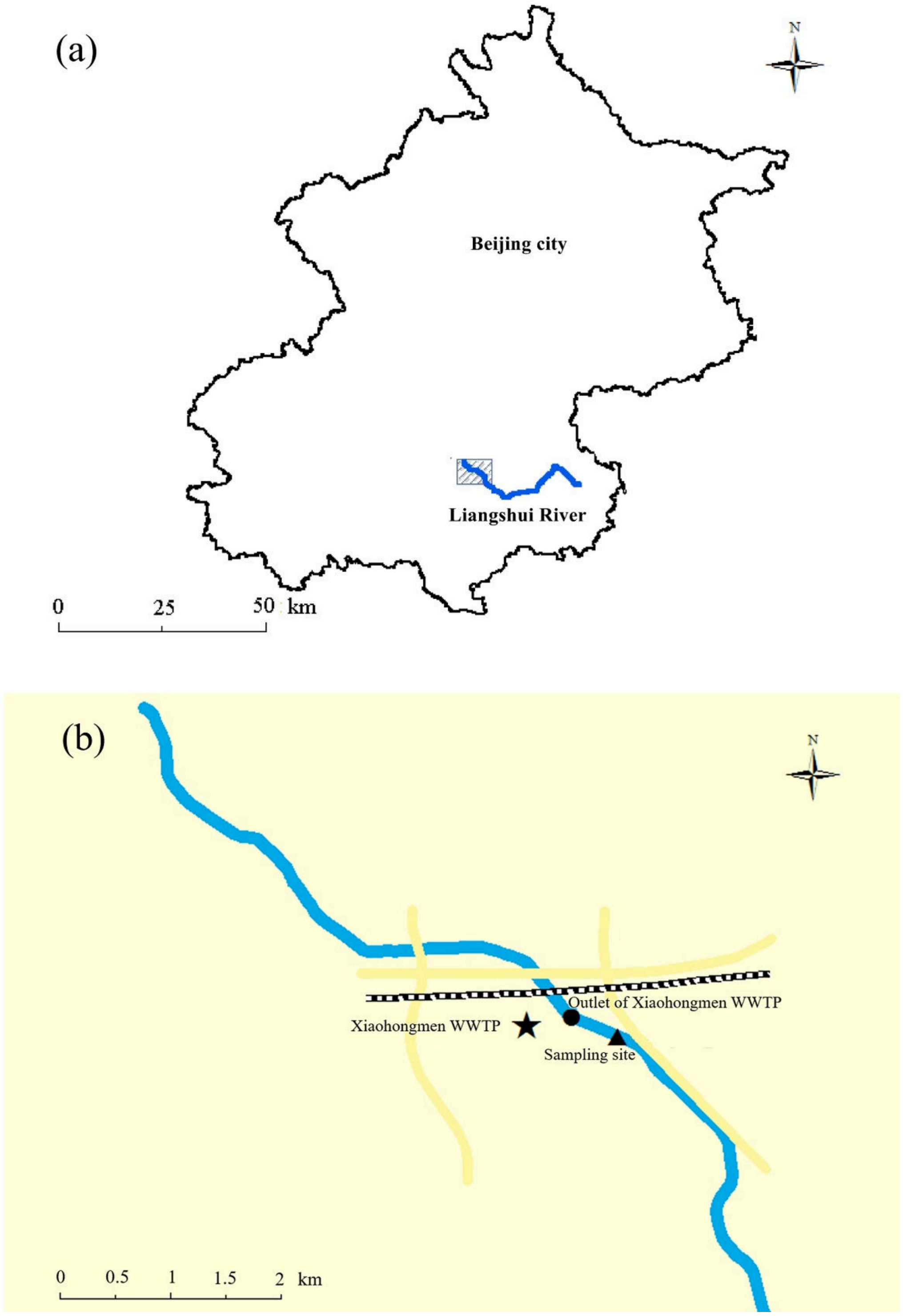


**Figure S1. (a) The location of Liangshui River in Beijing City, and (b) the location of Xiaohongmen WWTP (★), outlet of Xiaohongmen WWTP (●) and sampling site (▲).** The pictures were created by ArcMap10.3.

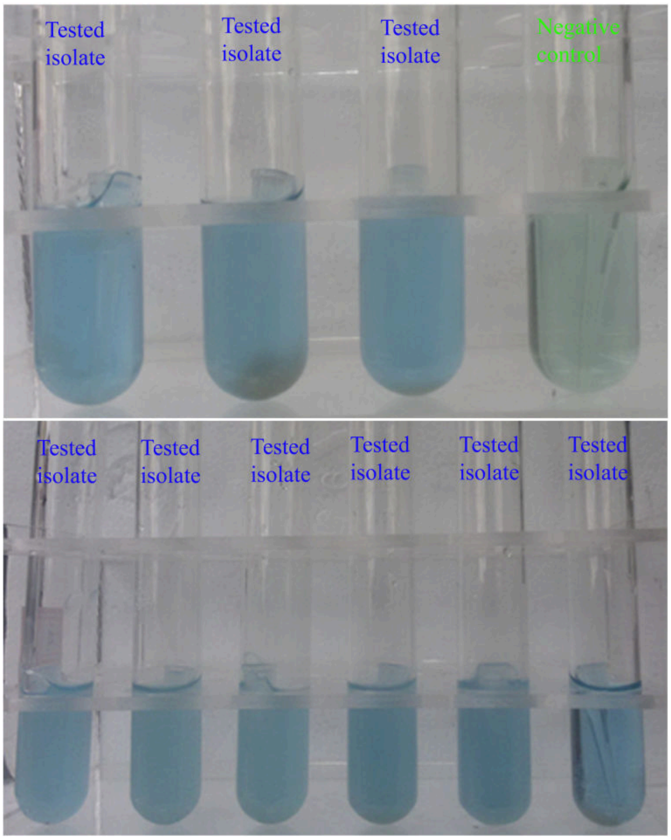


**Figure S2. Screening of the aerobic denitrifying bacteria in liquid screening media**. The SM containing only BTB was shown as green at pH of 7 (negative control), while the SM containing aerobic denitrifiers were shown as blue (Tested isolate).

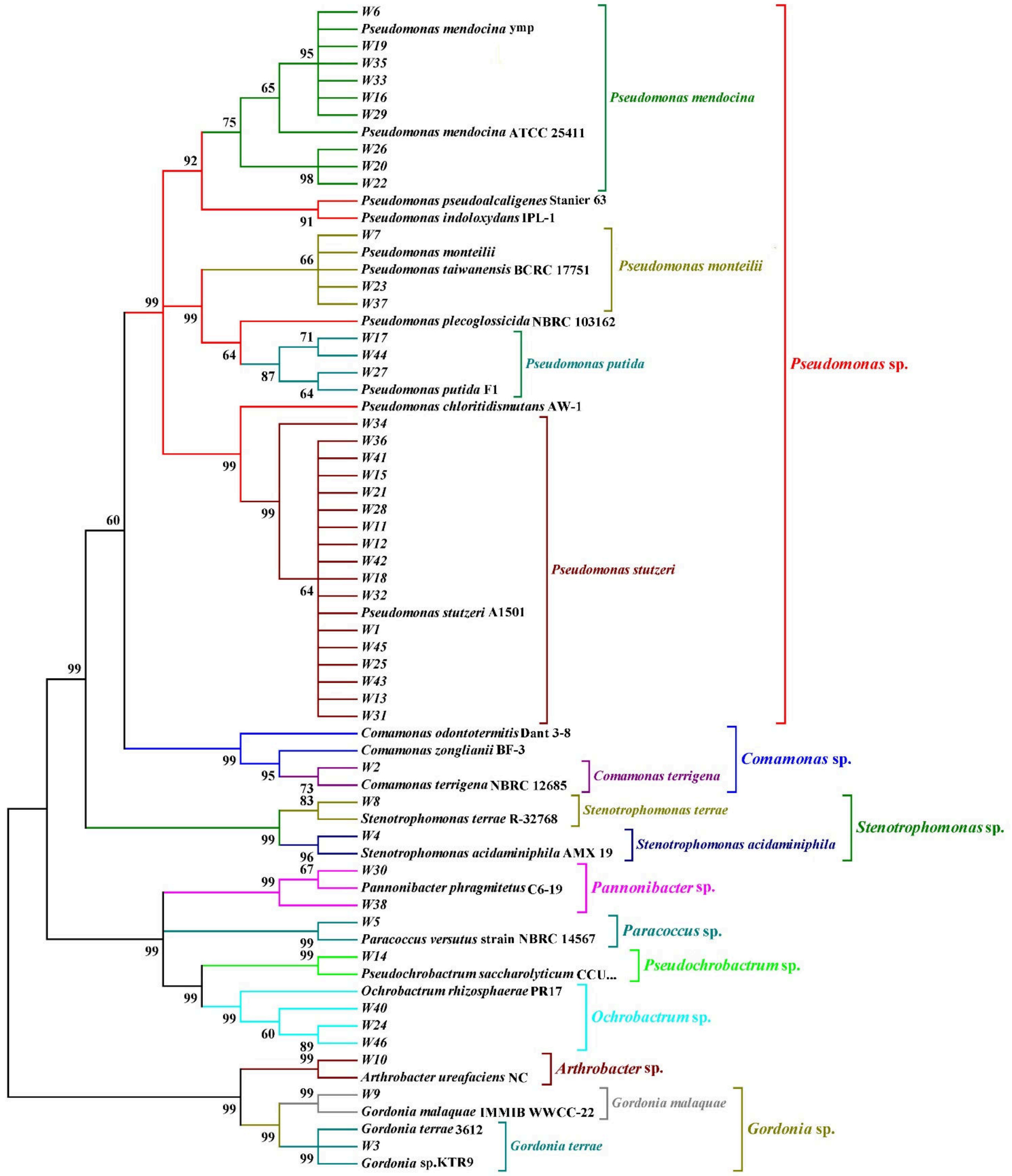


**Figure S3. The phylogenetic tree (rectangular mode) for the culturable aerobic denitrifying isolates in the water phase of Liangshui River**. The numbers next to the branches represent the bootstrap values.

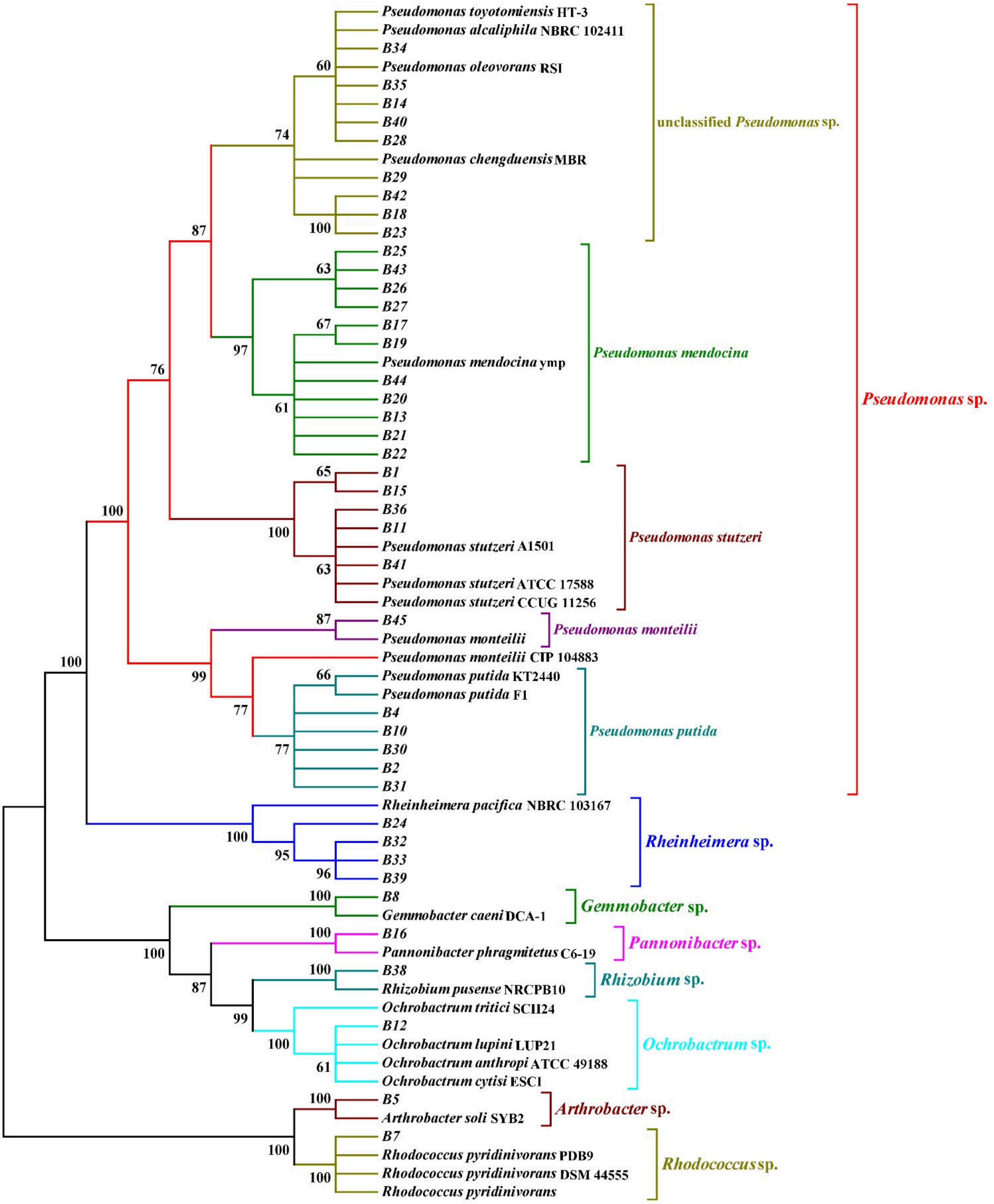


**Figure S4. The phylogenetic tree (rectangular mode) for the culturable aerobic denitrifying isolates in the biofilm phase of Liangshui River**. The numbers next to the branches represent the bootstrap values.

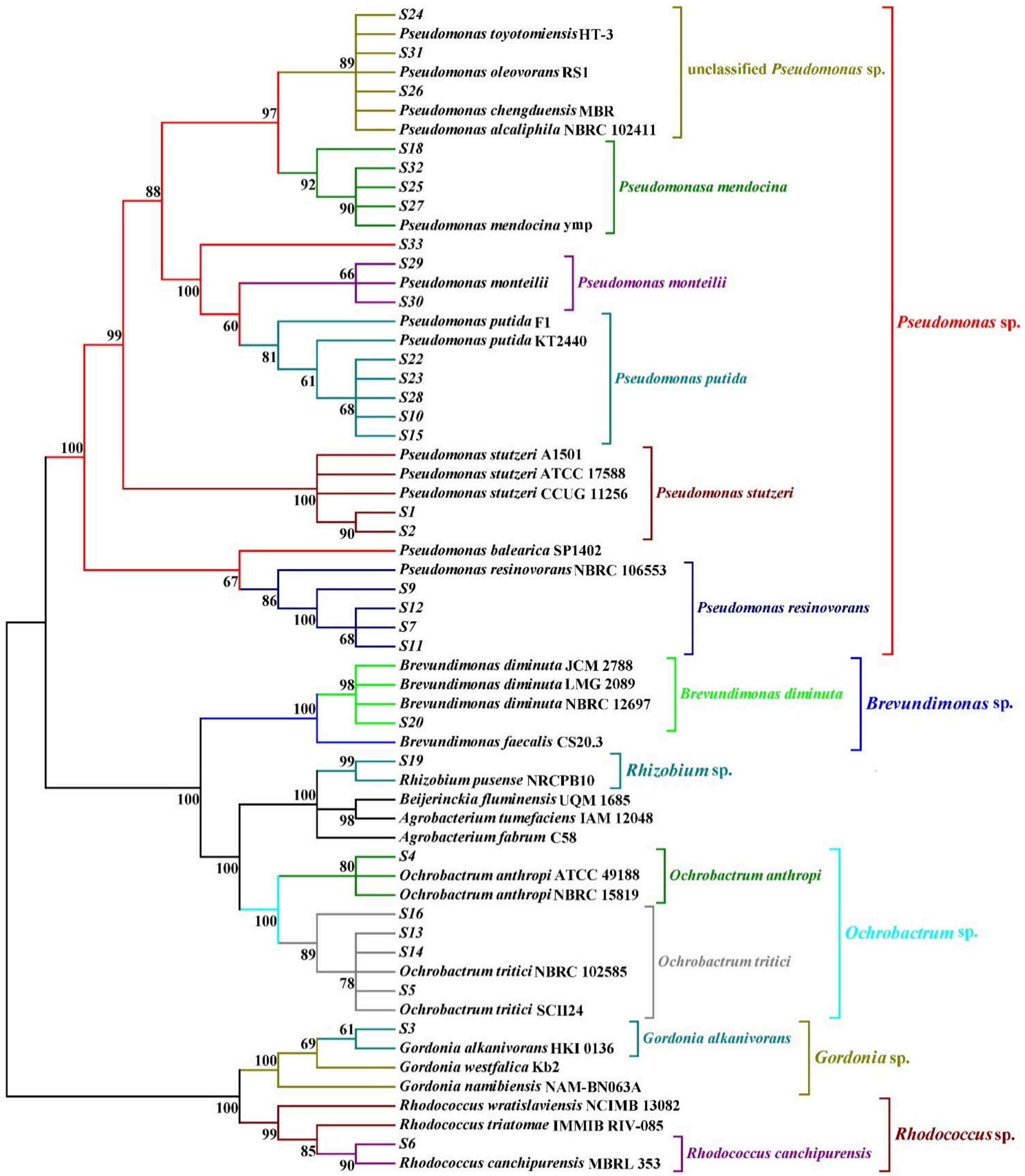


**Figure S5. The phylogenetic tree (rectangular mode) for the culturable aerobic denitrifying isolates from the sediment phase of Liangshui River**. The numbers next to the branches represent the boostrap values.

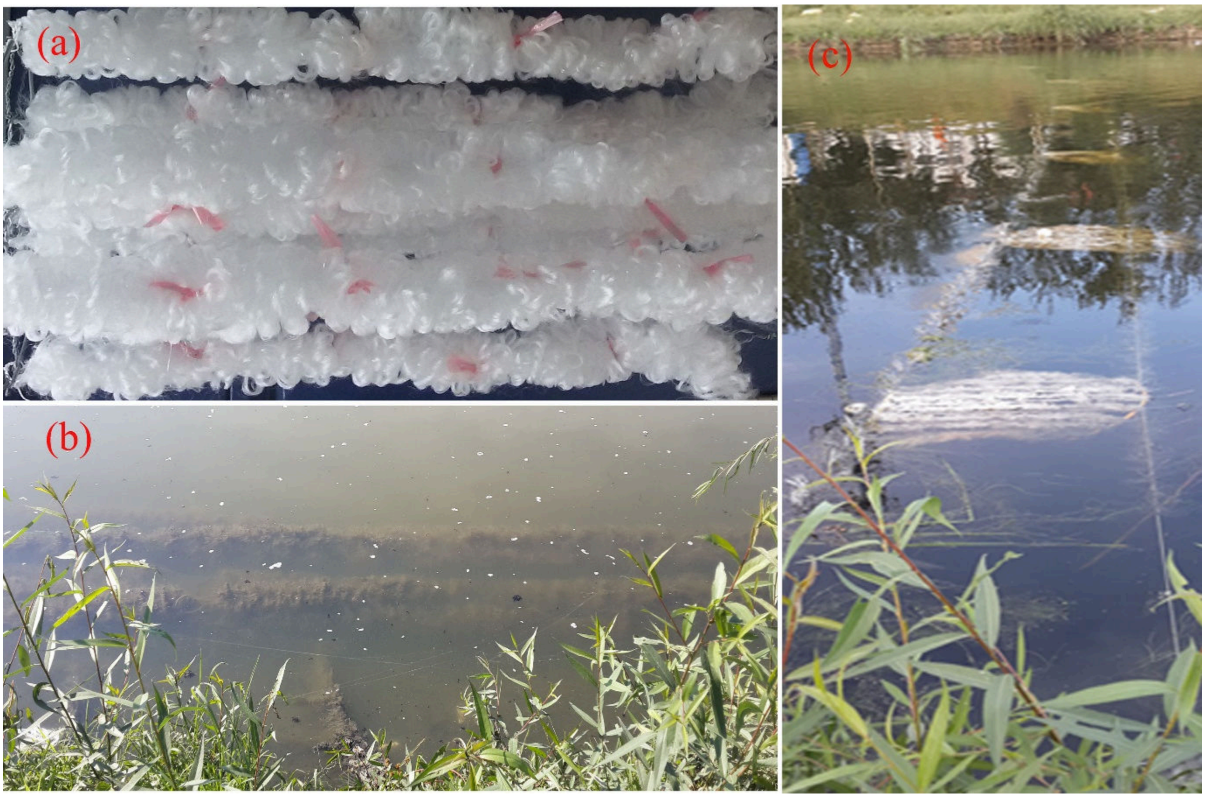


**Figure S6. The supporting materials used for the biofilm growth in the Liangshui River.** The image (a) shows the virginal materials while the pictures (b) and (c) represent the materials installed in the water phase of the Liangshui River.
